# Supplementary material for: Reversible morphology-resolved chemotactic actuation and motion of Janus emulsion droplets
Source: Nat Commun. 2022 May 10;13:2562. doi: 10.1038/s41467-022-30229-3 (PMC9091213; doi:10.1038/s41467-022-30229-3)
Supplement: Supplementary file 1 — Supporting Information [file 41467_2022_30229_MOESM1_ESM.pdf]

Supporting Information

Reversible morphology-resolved chemotactic actuation and motion of Janus emulsion droplets

Bradley D. Frank[1], Saveh Djalali[1], Agata W. Baryzewska[1], Paolo Giusto[1], Peter H. Seeberger[2], and Lukas Zeininger[1,*]

[1] Department of Colloid Chemistry, Max Planck Institute of Colloids and Interfaces, Am Muehlenberg 1, 14476 Potsdam, Germany; lukas.zeininger@mpikg.mpg.de

[2] Department of Biomolecular Systems, Max Planck Institute of Colloids and Interfaces, Am Muehlenberg 1, 14476 Potsdam, Germany

Contents

Supplementary Figure 1. Cell for the observation of droplet chemotaxis via linear surfactant gradients 2

Supplementary Figure 2. Droplet and particle tracking..3

Supplementary Figure 3. Emulsion droplet chemotaxis in response to surfactant concentration gradients 4

Supplementary Figure 4. Droplet stabilization and internal morphologies 5

Supplementary Figure 5. PIV of flow fields surrounding Janus droplets in three different morphologies.. 6

Supplementary Figure 6. Complex droplet volume ratio. 7

Supplementary Figure 7. Calibration curve for monodisperse droplet morphologies generated in AOT : Zonyl using microfluidics 8

Supplementary Figure 8. Calibration curve for microfluidic droplets generated in SDS : Zonyl 9

Supplementary Figure 9. Calibration curve for AzoTAB : Zonyl...10

Supplementary Figure 10. Directed lighting setup for initiating droplet movement via light-induced surfactant isomerization of Azo-TAB surfactants...11

Supplementary Figure 11. Droplet movement via light-induced interfacial gradients...12

Supplementary Figure 12. Enzymatic cleavage of β-n-octyl-galactopyranoside surfactants. 13

Supplementary Figure 13. Time dependence of the Janus droplet morphological response to surfactant gradients. 14

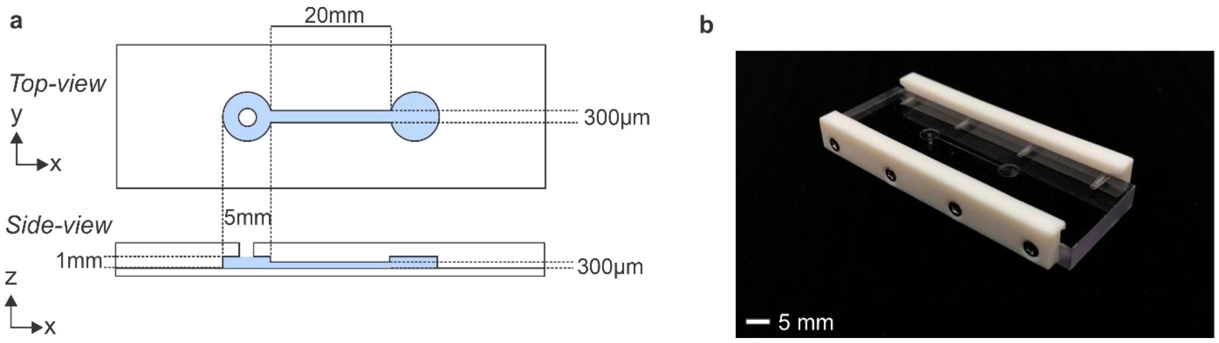

Supplementary Figure 1. Cell for the observation of droplet chemotaxis via linear surfactant gradients. a) Schematic including relevant dimensions and measurements of the milled microfluidic channel utilized in this study, drawing not to scale; b) Image of the polycarbonate microfluidic device used for the study of droplet movement in this study, where a glass slide is inserted into the tracks (white) to seal the droplets, before holder inversion for study.

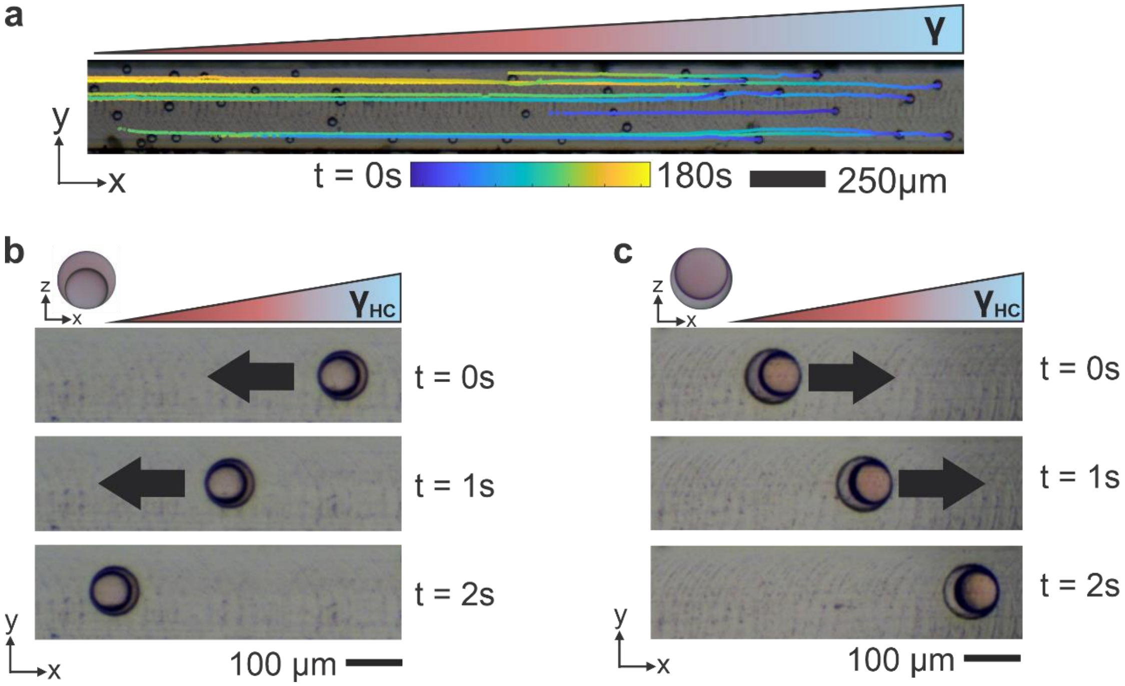

Supplementary Figure 2. Droplet and particle tracking; a) Optical micrograph of decane:bromohexane droplets in 0.1 WT % AOT tracked in the channel in response to added 1 WT % AOT; b) Optical micrograph of decane : methoxyperfluorobutane droplets with a hydrocarbon-dominant morphology in 1 WT % AOT : Zonyl responding to the addition of 1 WT % AOT, moving toward the added probe, as the imaging mode used for data collection; c) Optical micrograph of decane : methoxyperfluorobutane droplets with a fluorocarbon-dominant morphology in 1 WT % AOT : Zonyl responding to the addition of 1 WT % AOT, moving away from the added probe, as imaged for data collection.

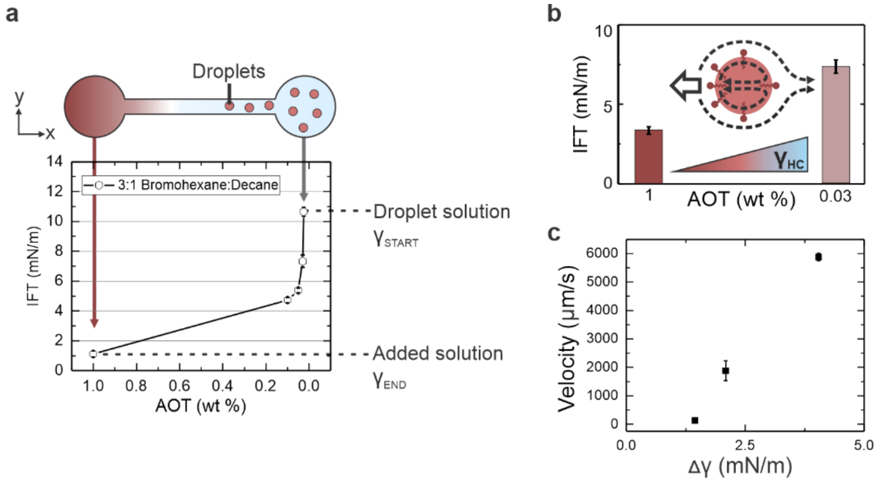

Supplementary Figure 3. Emulsion droplet chemotaxis in response to surfactant concentration gradients; a) Schematic drawing of the channel used for linear surfactant gradient delivery in this study depicting the addition of pure 1 WT % AOT to the left side of the chip, with an arrow pointing to the measured interfacial tension at that point, compared to the interfacial tension of droplets in their original solution; b) Graph with droplet drawing indicating the chemotactic reaction to the interfacial tension gradient; c) Velocity versus change in interfacial tension between the original and added surfactant for droplets of 3:1 bromohexane : decane, reacting to the linear interfacial tension gradient generated inside the channel, error bars denote standard deviation of n = 5 measurements.

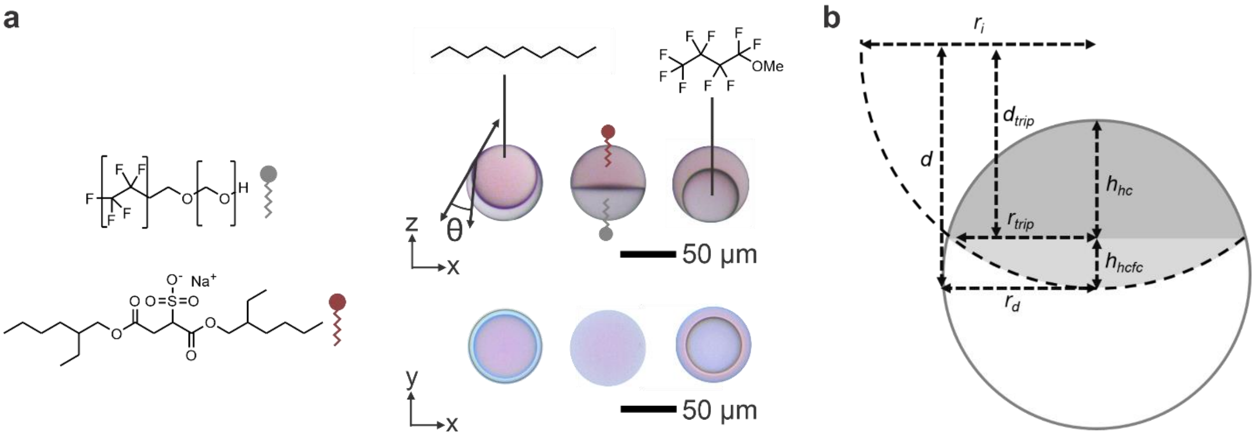

Supplementary Figure 4. Droplet stabilization and internal morphologies. a) Schematic for the calculation of the triple phase contact angle, the constituent droplet phases of decane and methoxyperfluorobutane, for which AOT and Zonyl (structures shown) are stabilizing, and sideview optical micrographs and matching optical micrographs of complex droplets in three varying morphologies, indicating the view and connection between droplets in side-profile (Z-X) and top-profile (X-Y); b) Schematic for the calculation of droplet contact angles, volume ratios, and Janus ratios from side view optical micrographs of gravity-aligned droplets based on the Neuman construction.

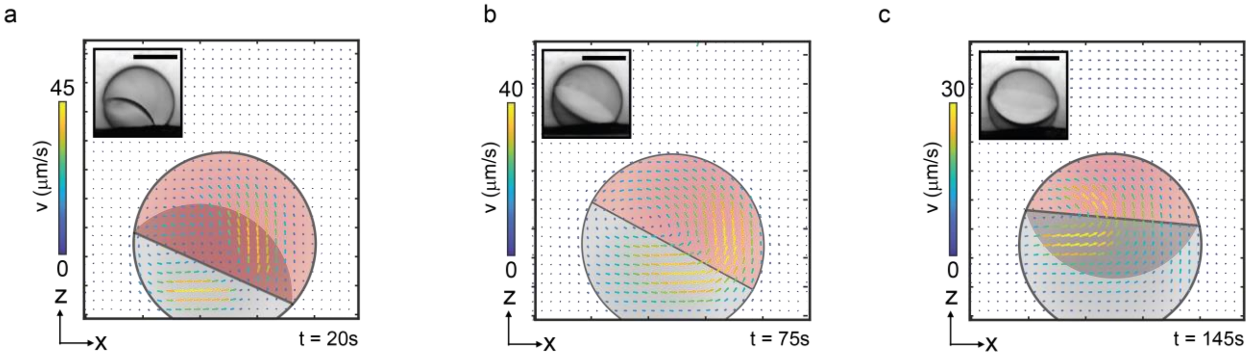

Supplementary Figure 5. PIV of flow fields surrounding Janus droplets in three different morphologies. Immobilized droplet composed of MPFB : Zonyl dispersed in a solution of AOT : Zonyl placed within the channel, with a 1:9 solution of 1 WT% AOT:Zonyl added to the channel. PIV tracking of tracer particles allows the visualization of the flow profiles surrounding the droplets, on the fluorocarbon interface travelling from –x to +x, as the gradient would indicate, and for the hydrocarbon interface flows travel from +x to –x (Supplementary Movie 3). a) 30s after initial droplet movement; b) 90s after initial droplet movement; c) 150s after initial droplet movement, scale bar = 50 μm.

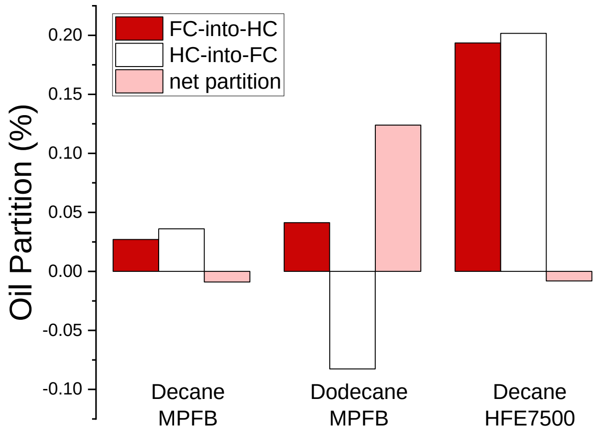

Supplementary Figure 6. Complex droplet volume ratio. Partitioning of oil into either phase of three different solvent combinations, determined via measuring the refractive index of oils before and after mixing above and below the upper critical solution temperature. Net partition represents the difference between partitioning into the hydrocarbon phase and the fluorocarbon phase.

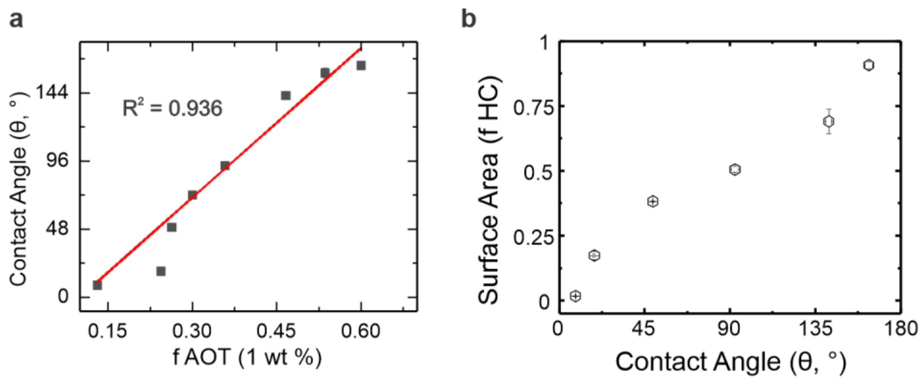

Supplementary Figure 7. Calibration curve for monodisperse droplet morphologies generated in AOT : Zonyl using microfluidics; a) Contact angle of decane : methoxyperfluorobutane droplets in various fractions of 1 WT % AOT : Zonyl, for the droplets used in this study. B) Plot of the ratio of exposed surface areas for droplets in different Janus morphologies expressed as the fraction of the HC surface area of the overall droplet surface area $f_{HC,}$ error bars denote standard deviation of n = 5 measurements.

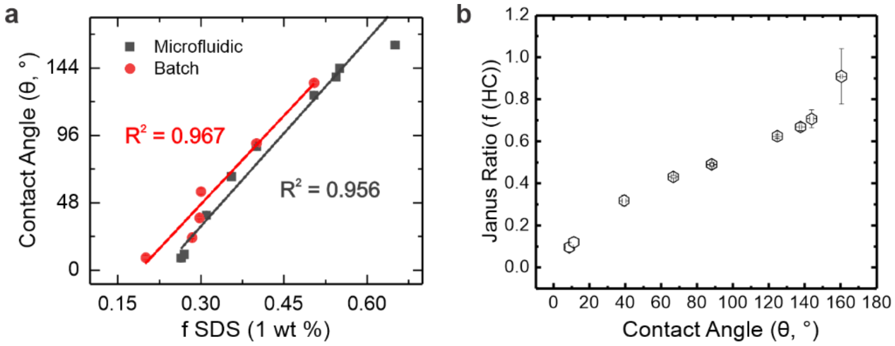

Supplementary Figure 8. Calibration curve for microfluidic droplets generated in SDS : Zonyl; a) Contact angle of decane : methoxyperfluorobutane droplets in various fractions of 1 WT % SDS : Zonyl, for the droplets used in this study, where the contact angles of droplets made directly in the surfactant solution is in red, and the contact angles of phase-transferred droplets in gray; b) Janus ratio as the fraction of the hydrocarbon surface area for methoxyperfluorobutane : decane droplets in SDS : Zonyl, error bars denote standard deviation of n = 5 measurements.

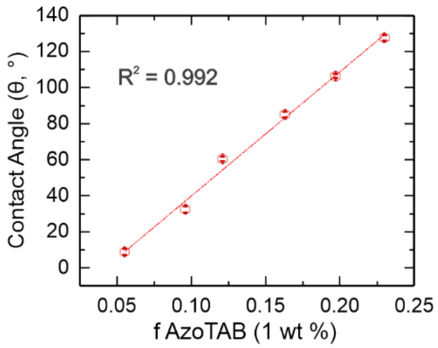

Supplementary Figure 9. Calibration curve for AzoTAB : Zonyl; Contact angle versus weight percentages of AzoTAB with constant 0.1 WT % Zonyl for methoxyperfluorobutane droplets utilized in this study. AzoTAB was kept in darkness until measurement with minimal illumination, error bars denote standard deviation of n = 5 measurements

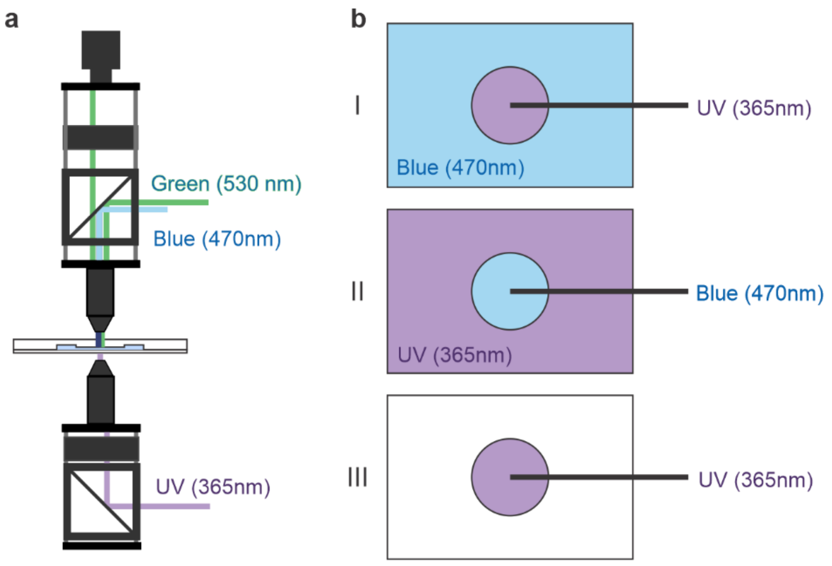

Supplementary Figure 10. Directed lighting setup for initiating droplet movement via light-induced surfactant isomerization of Azo-TAB surfactants. a) Schematic of the lighting setup utilized to deliver light either in full frame, or as point source to the droplet layers, featuring (top to bottom) CCD (HIKVision), tube lens (200mm, ThorLabs), filter cube (ThorLabs), objective (4x, 10x, 20x, Olympus), sample holder, and for light delivery a second objective, tube lens, and filter cube. All light delivered to the sample was collimated. A bifurcated fiber was used to deliver the full frame light of both green light (530nm, for viewing), and either blue or UV light. Green light delivered was constant and unchanged based on experimental conditions. Directed light was delivered to the bottom of the sample, with its own XYZ stage, allowing focus, alteration of spot size, and translation of the light source over the sample. This setup is mounted on a fully rotating angular stage, allowing the application of these lighting conditions both inverted and in side view to the same sample; b) diagram for the various lighting modes utilized for the study of AzoTAB. For most experiments, constant blue light (470nm) was delivered over the entire sample, and UV light was delivered in a spot (365nm), generating a spot, or zone of high interfacial tension. Alternatively (II) a blue spot is delivered, generating a zone of low interfacial tension. Finally in (III) UV light was delivered in a spot alone, changing the droplet morphology as well as inducing movement.

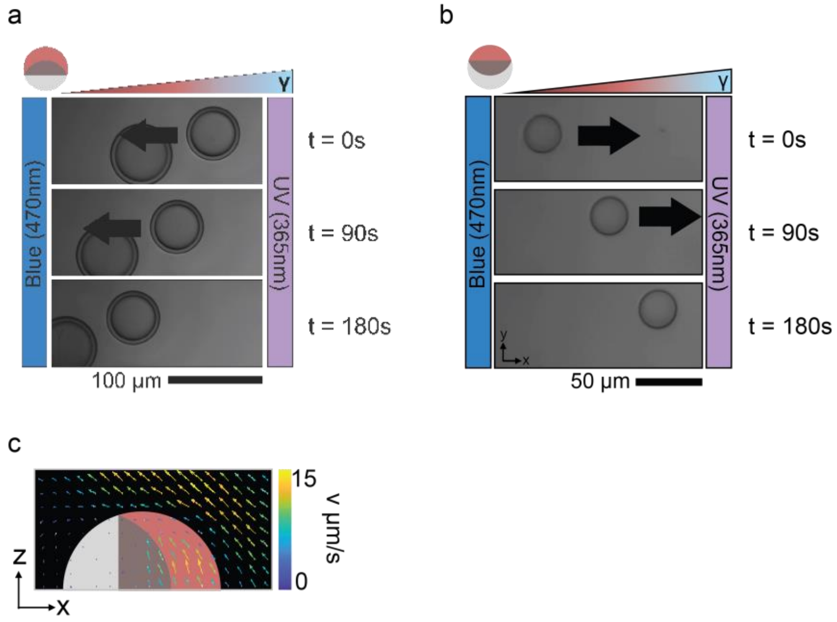

Supplementary Figure 11. Droplet movement via light-induced interfacial gradients. a) decane : methoxyperfluorobutane droplets stabilized by AzoTAB : Zonyl (0.23 WT% AzoTAB 0.1 WT% Zonyl, θ = 127º) in a hydrocarbon-dominant morphology move with respect to the dominant hydrocarbon phase when the sample is exposed to blue light (460nm, LED), and anisoptroic UV light (365nm, LED) on the right hand side; b) when decane : methoxyperfluorobutane droplets are generated in AzoTAB : Zonyl (0.1 WT% AzoTAB 0.1 WT% Zonyl, θ = 32º) with fluorocarbon-dominant morphologies, on the application of the same light source, droplets travel in the direction of the UV (365nm, LED) light source. C) PIV tracking of flow fields sourrounding a Janus droplet upon anisotropic UV-light application in the vicinity of the droplet.

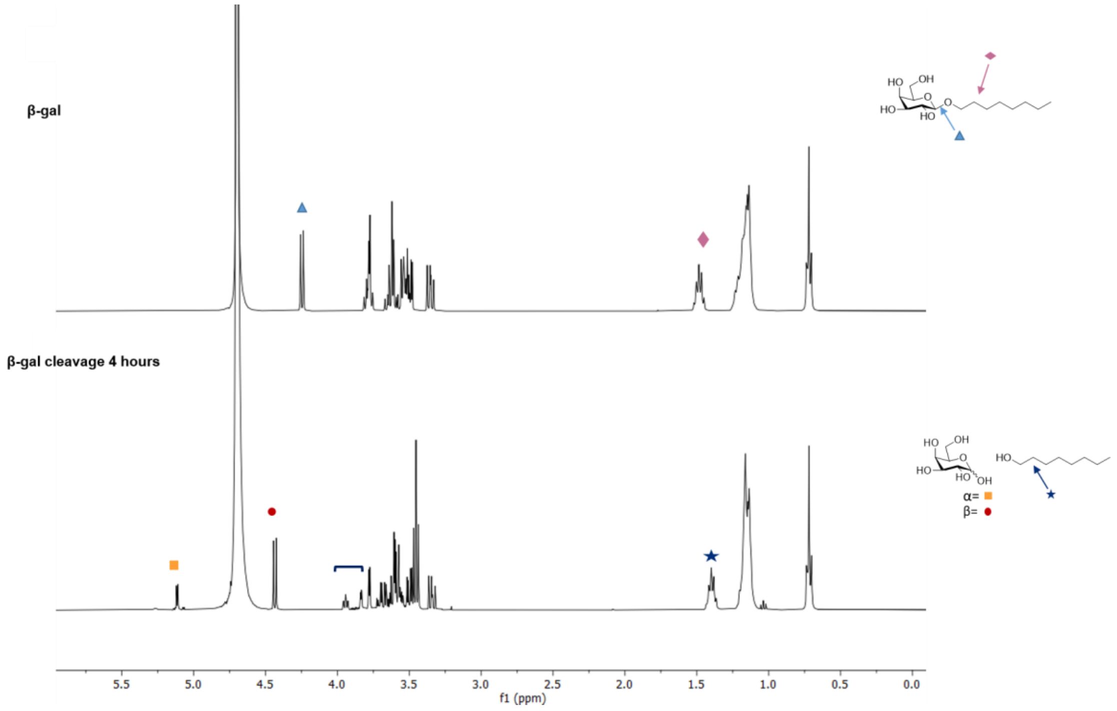

Supplementary Figure 12. Enzymatic cleavage of *β*-n-octyl-galactopyranoside surfactants. a) Time dependent NMR direct enzymatic cleavage study for the cleavage of *β*-n-octyl-galactopyranoside (0.1wt%) with β-galactosidase (1U/mL) in D2O.

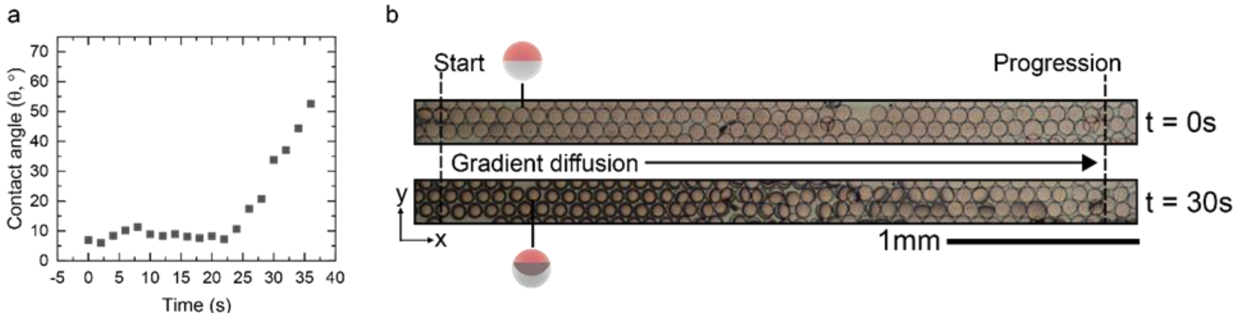

Supplementary Figure 13. Time dependence of the Janus droplet morphological response to surfactant gradients. a) Measurement of the time delay for the onset of a morphological transition of Janus droplets in response to changes in surfactant composition with respect to the instantaneous onset of chemotactic motion as a result of an evoked interfacial tension gradient; t = 0 marks the time at which freely moving Janus droplets began to move in response to an evoked surfactant gradient inside the channel. Inside the monitored section of the channel, we observed, in parallel to droplet motion, a droplet that was restrained from moving due to a defect in the channel and recorded the contact angle of this droplet at the same position over time.; b) Optical micrographs of Janus droplets dispersed inside a 1 wt.% surfactant solution of 4:6 SDS:Zonyl, placed as a densely packed monolayer inside the channel. Upon addition a 1 wt.% Zonyl solution to the inlet of the channel, the progression of the surfactant diffusion can be observed via droplet morphology changes throughout the channel. Dotted lines represent the progression of surfactant diffusion at t = 0s, and t = 30s respectively, (Supplementary Movie 10).
