# Supplementary material for: Reversible morphology-resolved chemotactic actuation and motion of Janus emulsion droplets
Source: Nat Commun. 2022 May 10;13:2562. doi: 10.1038/s41467-022-30229-3 (PMC9091213; doi:10.1038/s41467-022-30229-3)
Supplement: Supplementary file 3 — Description to Supplementary Information [file 41467_2022_30229_MOESM3_ESM.pdf]

Legends for Supplementary movies:

Supplementary Movie 1: Inverted microscope video (1x speed) of hydrocarbon-dominant droplets (decane : methoxyperfluorobutane in AOT:Zonyl (0.54:0.46 1 WT%)) moving in response to the addition of 1 WT % AOT.

Supplementary Movie 2: Sideview video (1x speed) of fluorocarbon-dominant droplets (decane : methoxyperfluorobutane in AOT:Zonyl (0.19:0.81 1 WT%) moving in response to the addition of 1 WT % AOT, with experimental setup and initial droplet morphology noted in video.

Supplementary Movie 3: Microscope video (side-view) of a hydrocarbon-dominant droplet of decane and methoxyperfluorobutane stabilized in AOT:Zonyl (0.42:0.58 1 WT%) responding to the addition of 1:9 AOT:Zonyl with added tracer particles to characterize the anisotropic flow profile.

Supplementary Movie 4: Inverted microscope video (1x speed) of hydrocarbon-dominant droplet (0.49:0.51 1 WT%) moving in response to the addition of 1 WT % Zonyl, with experimental setup and initial droplet morphology noted in video. Droplets initially move away from the added surfactant (left) before changing morphology, coming to a halt, and moving toward the added surfactant once the fluorocarbon phase was dominant.

Supplementary Movie 5: Inverted microscope video (1x speed) of Janus droplets (decane : methoxyperfluorobutane) in AzoTAB : Zonyl (0.18 WT% AzoTAB, 0.1 WT% Zonyl) under green (530nm) illumination responding to anisotropic illumination of UV light (indicated when applied), demonstrating surfactant diffusion, droplet movement, and tilting.

Supplementary Movie 6: Inverted microscope video (1x speed) of Janus droplets (decane : methoxyperfluorobutane) in AzoTAB : Zonyl (0.18 WT% AzoTAB, 0.1 WT% Zonyl) under green (530nm) and blue (470nm) illumination responding to anisotropic illumination of UV light (indicated by faint spot) with added tracer particles, demonstrating reconfiguration, tilting, and flows with the continuous balance of the switchable surfactant.

Supplementary Movie 7: Sideview microscope video (1x speed) of a hydrocarbon-dominant droplet (decane : methoxyperfluorobutane) in AzoTAB : Zonyl (0.23 WT% AzoTAB, 0.1 WT% Zonyl) under green (530nm) and blue (470nm) illumination, with added tracer particles. Under anisotropic illumination with UV light (365nm, indicated with purple spot), the induced flows due to interfacial tension gradients can be viewed. Initial droplet morphology and experimental setup are noted in the bottom left-hand corner.

Supplementary Movie 8: Microscope video (top-down, 5x speed) of Janus droplets (decane : methoxyperfluorobutane) in AzoTAB : Zonyl (0.1 WT% AzoTAB, 0.1 WT% Zonyl) under green (530nm) and blue (470nm) illumination responding to anisotropic illumination of UV light (indicated by purple spot). In this context, droplets with a fluorocarbon-dominant move toward the light source (as opposed to away from it). Initial droplet morphology and experimental setup are noted in the bottom left-hand corner.

Supplementary Movie 9: Microscope video (top-down) of hydrocarbon- and fluorocarbon- dominant Janus droplet, 0.5 WT % *β*-n-octyl-galactopyranoside with varying Zonyl concentrations (0.05 WT% for droplets with (θ = 162°), and 0.1 WT% Zonyl for droplets with (θ = 7°), composed of decane and methoxyperfluorobutane moving in response to the addition of 1 µL of a surfactant solution containing *E. coli* bacteria at 8 10^8 CFU mL^{-1}.

Supplementary Movie 10: Microscope video of Janus droplets dispersed inside a 1 wt.% surfactant solution of 4:6 SDS:Zonyl, placed as a densely packed monolayer inside the channel. Upon addition a 1 wt.% Zonyl solution to the inlet of the channel, the progression of the surfactant diffusion can be observed via droplet morphology changes throughout the channel.
